# A comparative study on monitoring of bioactive compound production/degradation, volatile substances, and oxidation indices during horn and bath high-power ultrasound-assisted oil bleaching

**DOI:** 10.1016/j.ultsonch.2024.107184

**Published:** 2024-11-30

**Authors:** Mehran Sayadi, Elahe Abedi, Malihe Keramat

**Affiliations:** a Department of Food Safety and Hygiene, Faculty of Health, Fasa University of Medical Sciences, Fasa, Iran; b Department of Food Science and Technology, Faculty of Agriculture, Fasa University, Fasa, Iran

**Keywords:** Volatile substances, Tocopherol degradation/adsorption, Ultrasonic-assisted bleaching, Sunflower oil

## Abstract

This research aimed to investigate and compare the effect of bath and horn ultrasound-assisted bleaching of sunflower oil on the degradation of tocopherols and sterols, production of volatile substances, and oxidation indices, including thiobarbituric acid (TBA) and peroxide value (PV) and with that of the industrial bleaching process. Ultrasonic bath and ultrasonic horn bleaching techniques reduced sunflower oil’s total tocopherol and total sterol contents to a greater extent than conventional bleaching techniques. While bath and horn sonication operated theoretically equivalent power settings, power meter measurements demonstrated that the bath sonicator delivered significantly less power than the horn sonicator. Among the ultrasonic bleaching techniques, the ultrasonic bath at 400 W showed the lowest reduction in total tocopherols, sterols and volatile compounds compared to the ultrasonic horn technique at the same theoritical power. Moreover, Despite the 800 W bath sonicator having significantly higher nominal power than the 400 W horn sonicator, the horn sonicator was considerably more effective at degrading bioactive compounds. Higher degradation of bioactive compounds coincided with increasing patterns in primary and secondary oxidation indices and volatile compounds in horn compared to bath and industrial bleaching due to the direct effect of ultrasonic horn and free radical formations.

## 1 Introduction

Bleaching is one of the significant steps of vegetable oil refining. In conventional bleaching, activated carbon or bleaching clay removes impurities and undesirable color pigments from the oil. Mineral acids (e.g., sulfuric acid and hydrochloric acid) are added to the bleaching clays to increase the surface area. The bleaching time, type and amount of bleaching clay, and temperature can impact the efficiency of the bleaching process. Generally, the conventional bleaching technique is performed at high temperatures (90–120 ◦C) for 20–30 min. The amount of clay used for bleaching is about 0.5–2 % [1]. Some unfavorable side reactions can occur during conventional bleaching, which results in the formation of the cyclic polymer, increasing free fatty acid contents, and cleavage of oxidation products [2]. Besides, a significant amount of oil is loosed during conventional bleaching [3]. Enhancing the amount of clay can cause higher removal of color pigments but also result in higher oil loss and environmental problems. Furthermore, applying high amounts of acid-activated clay results in the production of oxidation products [4]. Other disadvantages of conventional bleaching are the high cost of bleaching clay and the time-consuming process of bleaching clay removal from the oil [5], [6]. In recent years, novel techniques such as supercritical fluid, membrane technology, high-voltage electric field, and ultrasound techniques have been investigated as alternatives to conventional bleaching techniques [7], [8], [9], [10]**.**

Applying the ultrasound technique for oil bleaching has several benefits, such as lower processing time and temperature, higher efficiency, improved oil yield, low energy consumption, and lower clay utilization, and saved cost, compared to the conventional technique [11], [12].

In the ultrasound technique, the cavitation phenomena can result in more efficient bleaching and improve the mechanism of physical adsorption, mass transport, and fluid turbulence. Besides, higher adsorptive sites are created between solid and liquid boundaries, improving bleaching efficiency and decreasing bleaching clay consumption, costs, oil loss, and processing time [12], [13], [14], [15], [16]. It has been reported that bleaching by ultrasound technique did not cause changes in *cis*-fatty acid configuration [17].

Bath and horn systems are two significant types of ultrasound devices. An ultrasonic bath device includes a metallic vessel equipped with a piezoelectric transducer. The transducers generate vibrations that propagate significant types of ultrasound devices. Ultrasonic bath device consists of a metallic vessel equipped with a piezoelectric transducer. The transducers generate vibrations propagating throughout the vessel and into the liquid medium in the tank. This device distributes the ultrasonic waves uniformly within the liquid medium. The main advantages of applying ultrasonic bath devices in the food industry are low cost, scale-up, and user-friendliness.

Besides, ultrasonic bath devices are readily available and can operate continuously. Although ultrasonic bath devices have several benefits, they have some limitations, which can cause poor reproducibility and repeatability of this device. Uneven temperature distribution in the bath is one of the limitations of ultrasonic bath devices. Another limitation of this device is difficulty controlling essential process parameters such as frequency and power output. In an ultrasonic horn device, the ultrasonic energy is localized on the sample zone, generating more efficient cavitation in the liquid compared to that of an ultrasonic bath [18]. In addition, the ultrasonic horn device can apply a higher ultrasound intensity to the sample container and independence from distance than an ultrasonic bath. The main disadvantages of the ultrasonic horn device are its high cost and scale-up [19], [20].

Tocopherols and sterols are two major groups of bioactive compounds in sunflower oil. Tocopherols, commonly referred to as vitamin E, are recognized as potent antioxidant molecules that occur naturally in vegetable oils. There are four forms of tocopherols: α-, β-, γ-, and δ-tocopherol. Among these, γ-tocopherol has demonstrated the highest in vitro antioxidant activity, followed closely by δ-tocopherol [21]. Sunflower oil is rich in phytosterols, with β-sitosterol being the predominant sterol. The content of phytosterols is primarily influenced by environmental conditions during plant growth and genetic factors; however, modifications to the fatty acid profile do not significantly affect phytosterol levels [4].

The main objective of vegetable oil bleaching is to minimize the bioactive compounds degradation and vegetable oil loss while eliminating undesirable impurities as much as possible [4]. Some studies have investigated the application of ultrasonic baths and horns for the bleaching of soybean oil, canola oil, olive oil, sunflower oil, palm oil, and olive oil [9], [13], [14], [15], [16]. However, no study has compared the effect of an ultrasonic bath device with that of an ultrasonic horn device on the degradation of bioactive compounds and the formation of oxidation products during vegetable oil bleaching. Hence, this research aimed to compare sunflower oil's ultrasonic bath with horn bleaching containing carotenoid. Changes in bioactive compounds (tocopherols and sterols), volatile oxidative compounds, fatty acid composition, and oxidation indices of sunflower oil during bleaching by ultrasonic bath and horn devices at different powers were compared with conventional technique.

## 2 Materials and methods

### 2.1 Materials

All chemical compounds applied in this research were of analytical grade with the highest purity and received from Merck (Darmstadt, Germany).

### 2.2 Bleaching process

Sunflower oil was purchased from Narges Oil Company (Shiraz, Fars Province). The bleaching process involved experiments with activated bentonite clay incorporated into sunflower oil (50 mL). Ultrasonic horn and ultrasonic bath devices were applied to bleach sunflower oil using ultrasound. An ultrasonic cleaning bath from Pacisa SA in Spain was utilized. The working power and the frequency were 1000 W and 25 kHz, respectively. The ultrasonic processor is a rectangular chamber measuring 30 cm × 15 cm × 15 cm, and a temperature controller monitors the temperature. Bleaching by ultrasonic bath device was conducted at two amplitudes [40 % (BU400) and 80 % (BU800) W]. For ultrasonic horn bleaching, the oil samples (50 mL) were subjected to sonication using an ultrasonic generator of the UP400S Hielscher type. The generator frequency and power output were 24 kHz and 400 W, respectively. An immersible horn was positioned in a 100-mL cylindrical jacket glass vessel. The horn was immersed in the liquid at the top of the vessel, transmitting sound vibrations into the oil sample through a titanium alloy rod with a diameter of 14 mm. The ultrasonic horn bleaching was conducted at two amplitudes [50 % (HU200) and 100 % (HU400)]. The effect of various bleaching parameters was evaluated by altering the levels of ultrasonic power (200 and 400 W for ultrasonic horn and 400 and 800 W for ultrasonic bath), temperature (35, 45, 55, and 65 °C), and bentonite clay percentage (0.5 %, 1 %, and 1.5 %).

In the theoretical approach, the same power was used. The bath UAB of oils was conducted at two amplitudes (40 % and 80 % W). However, in a practical approach, using a calorimetric method, the right power delivered was measured to be approximately 261 ± 2 W (40 %) and 624 W (80 %). This method involves the following equation:

$$\text{P}\;\left(\text{W}\right)=\text{m Cp}\;\left(\text{dT}/\text{dt}\right),$$

where m denotes the mass of liquid (kg), Cp is the liquid heat capacity (J/kgK), and (dT/dt) shows the rate rise in the temperature (K/s).

The industrial bleaching process conducted without ultrasonics served as the control. For the industrial bleaching process, the amount of bleaching clay was 1.5 %, the bleaching temperature was 120 °C, and the bleaching time was 30 min. All tests were performed in triplicate.

### 2.3 Physico-chemical Properties

The peroxide value (PV) was measured using the AOCS method Cd 8–53 [22]. Thiobarbituric acid (TBA) was determined by measuring the absorbance at 532 nm using the method of Rehab and El Anany. [23].

### 2.4 Measuring sterols

AOCS method Ce 7–87 was applied for measuring sunflower oil sterols. To prepare trimethylsilyl derivatives of the sterols, 100 µL bis(trimethylsilyl)-triuoro-acetamide was mixed with trimethylchlorosilane (1 %) and pyridine. Then, the mixture was heated at 60˚C for 0.5–1 h. An Agilent (Little Falls, DE, USA) device was applied for gas chromatography assay. A split-splitless injector, a SPB-5 column (30 m × 0.25 mm i.d., 0.25 µm of film thickness, Supelco, Inc. Bellefonte, PA), and a flame ionization detector were applied for gas chromatography assay. Helium was applied as carrier gas with 20 cm/s flow rate. The injection volume was 1.0 µL, the split ratio was 1:22, and the injector and detector temperatures were 295 ˚C and 300 ˚C, respectively. The column temperature was started at 265˚C and increased to 300 ˚C during 35 min. The column temperature was kept at 300 ˚C for 5 min. The retention times were compared to those of the sterol standards to identify the peaks. α-Cholestanol (5–750 mg) was used as an internal standard for quantification of all sterols [22].

### 2.5 Measuring tocopherols

The AOCS method Ce 8–89 was used for measuring tocopherols of sunflower oil. A normal phase high-performance liquid chromatography (HPLC) (Agilent Technologies, L1200) was used for measuring tocopherols. The HPLC device had a UV–Vis detector and a YMC-Pack SIL silica column (250 × 46 mm i.d. and 5 µm particle size). Acetonitrile/methanol/water (5:47.5:47.5 v/v) was applied as a solvent for tocopherol elution. The detection wavelength and the solvent flow rate were 292 nm and 1 mL/min flow rate, respectively. The injection volume was 20 µL. Before injection, sunflower oil was diluted with hexane (100 mL) and filtered by a 0.45 mm nylon syringe filter. To identify tocopherol isomers, the retention times were compared with those retention times of α, β, γ and δ-tocopherol standards. External calibration curves of tocopherol isomers were used for tocopherol quantification [22].

### 2.6 Determination of fatty acids composition

The fatty acid methyl esters (FAME) of sunflower oil samples were prepared by methylation of triacylglycerol, described by Abedi et al. (2015). The FAME were analyzed using a Shimadzu 17A (Kyoto, Japan) GC equipped with an FID and a fused silica capillary column (50 m × 0.25 mm and 0.20 µm of Carbowax 20 M). The column temperature was programmed at 2 °C/min from 150 to 240 °C. The injection port and detector temperature were maintained at 220 °C and 245 °C, respectively. The carrier gas was hydrogen (1.2 ml/min), the make-up gas was nitrogen (30 ml/ min), and the split ratio was 1:100. The FAME peaks were identified using FAME standards [13].

### 2.7 Determination of volatile compounds

Volatile compounds were determined using GC equipped with mass spectroscopy after a solid phase micro-extraction procedure (SPME), according to Jahouach-Rabai et al. (2008), with some modifications. Four mL of bleached sunflower oil were placed in a 10 mL headspace vial fitted with a silicone septum, which was placed in a water bath at 50 ˚C under magnetic stirring for 30 min. Volatile compounds were simultaneously trapped on an SPME fiber (divinyl benzene/carboxen/polydimethylsiloxane coating, Supelco, Bellefonte, PA). After trapping, volatile desorption occurs in a *cis*-4 PTV (Gerstel) injector at 250 ˚C. The separation of compounds began immediately. A Hewlett Packard 6890 GC/MS system is used for a chromatographic separation on an HP5-MS capillary column (30 m × 0.25 mm × 0.25 µm film thickness). GC analyses were performed using the following conditions: carrier gas He; flow rate 1.2 mL/min; injection temperature 250 ˚C; oven temperature programmed from 40 to 140 ˚C at 3 ˚C /min; then from 140 to 220 ˚C at 10 ˚C /min and holding at 220 ˚C; the ionization mode used was electronic impact at 70 eV. The components were identified from their linear retention indices, determined concerning a homologous series of alkanes, and by comparing their mass spectral fragmentation patterns with those stored in the data bank (Wiley/NBS).

### 2.8 Statistical analysis

One way to analyze variance was to determine significant differences among the mean values. Duncan’s multiple range test was used to compare the mean values (*P* < 0.05). The statistical software package SAS 9.1 (SAS Institute, Cary, NC) was used to analyze the experimental data and obtain the regression coefficients.

## 3 Results and discussion

### 3.1 Effect of ultrasonic bath and horn technique on sunflower oil oxidation

**Fig. 1:**
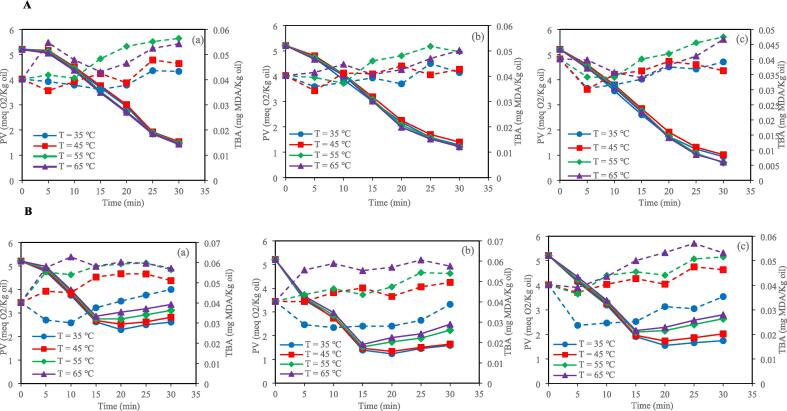

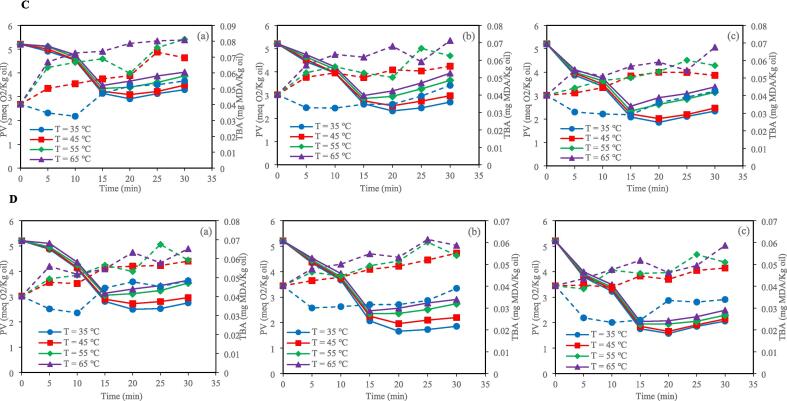

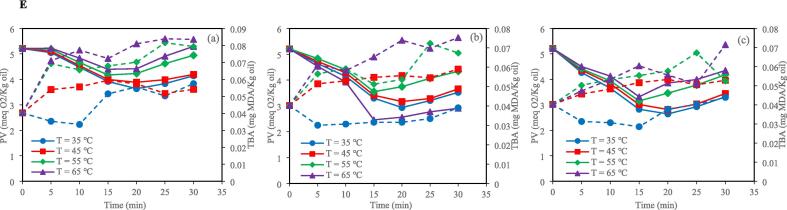
Changes of PV (▬) and TBA (‐‐) with time and temperature for (a) 0.5 %, (b) 1 %, and (c) 1.5 % clay under control conditions (A); Changes of PV (▬) and TBA (‐‐) with time and temperature for (a) 0.5 %, (b) 1 %, and (c) 1.5 % clay under HU_200_SU condition (B); Changes of PV (▬) and TBA (‐‐) with time and temperature for (a) 0.5 %, (b) 1 %, and (c) 1.5 % clay under HU_400_SU condition (C); Changes of PV (▬) and TBA (‐‐) with time and temperature for (a) 0.5 %, (b) 1 %, and (c) 1.5 % clay under BU_400_SU condition (D); Changes of PV (▬) and TBA (‐‐) with time and temperature for (a) 0.5 %, (b) 1 %, and (c) 1.5 % clay under BU_800_SU condition (E). HU_200_SU: ultrasonic horn (200 W), HU_400_SU: ultrasonic horn (400 W), BU_400_SU: ultrasonic bath (400 W), BU_800_SU: ultrasonic bath (800 W). The bleaching time is 30 min; IBSU is industrial bleaching, PV is peroxide value, TBA is thiobarbituric acid, and MDA is malondialdehyde.

According to SAS analysis, the PV and TBA values were significantly influenced by the interaction effect of bleaching time and temperature and ultrasonic bleaching mode (horn and bath) (Fig. 1). The PV is an indicator of primary oxidation products [23]. The results indicated that control and ultrasound bleaching decreased the PV in all investigated temperatures and clay concentrations. This decrease in PV can be attributed to the entrapment of lipid hydroperoxides by the bleaching clay [24]. By increasing the time of bleaching, the PV of the control bleached oil was consistently reduced, but in the ultrasonic horn and bath bleaching, this index decreased until 20 min and then increased. This increase in PV can be related to lipid peroxidation and forming free radicals, reducing bioactive compounds with antioxidant activity, and generating high local pressure and temperature during sonication [25], [26]. Fluctuations in PV can be linked to tocopherol levels. According to the results obtained by Abedi et al. (2015), ultrasound-assisted bleaching resulted in increases in γ-tocopherol and δ-tocopherol after the midpoint of the bleaching process. However, after 20 min of ultrasonic bleaching, tocopherol and sterol levels decreased, likely due to oxidative reactions. This reduced oil antioxidant activity and increased PV and TBARS values.

The TBA value is an indicator of secondary oxidation products [28]. In the control and ultrasonic techniques, sunflower oil samples bleached at higher temperatures (55 and 65 ˚C) showed higher TBA values than those bleached at lower temperatures (35 and 45 ˚C) after 30 min. Also, in both the ultrasonic bath and ultrasonic horn techniques, the TBA values of sunflower oil samples were increased by increasing power. In addition, after 30 min, the TBA value of sunflower oil samples bleached by the ultrasonic bath technique at 400 W was lower than that of sunflower oil samples bleached by the ultrasonic horn technique at the same power. Lipid oxidation during ultrasonic bath and ultrasonic horn techniques is related to cavitation. Cavitation is the formation, growth and, sometimes, the implosion of micro-bubbles formed in a liquid when ultrasound waves propagate through it. When the bubbles collapse, the energy accumulates in hot spots, the temperature reaches over 5000 ˚C, and the pressure reaches about 500 MPa. This phenomenon can result in peroxidation via three mechanisms, which can act alone or combined. The first mechanism is purely thermal, related to the high temperatures achieved during cavitation. The second mechanism is related to free radicals produced by sonolysis, and the third mechanism is related to the mechanical forces produced by shock waves and microstreaming [21]. In an ultrasonic bath device, the ultrasonic waves are distributed uniformly. In contrast, in an ultrasonic horn device, the ultrasonic energy is localized on the sample zone, generating more efficient cavitation in the liquid than in an ultrasonic bath [17]. Higher cavitation can result in a higher sunflower oil oxidation rate in the ultrasonic horn technique. Another reason for the higher oxidation rate during bleaching by the ultrasonic horn technique than that of the ultrasonic bath technique is the contact between the sunflower oil and the metallic ultrasonic horn, which can enhance the lipid oxidation rate [21].

There was no significant difference between the TBA values of sunflower oil samples bleached by ultrasonic bath technique at 400 W and those of sunflower oil samples bleached by ultrasonic horn technique at 200 W. Furthermore, the TBA values of sunflower oil samples bleached by ultrasonic bath technique at 800 W were similar to those bleached by ultrasonic horn technique at 400 W after 30 min.

### 3.2 Effect of ultrasonic bath and horn technique on the bioactive compounds

**Fig. 2:**
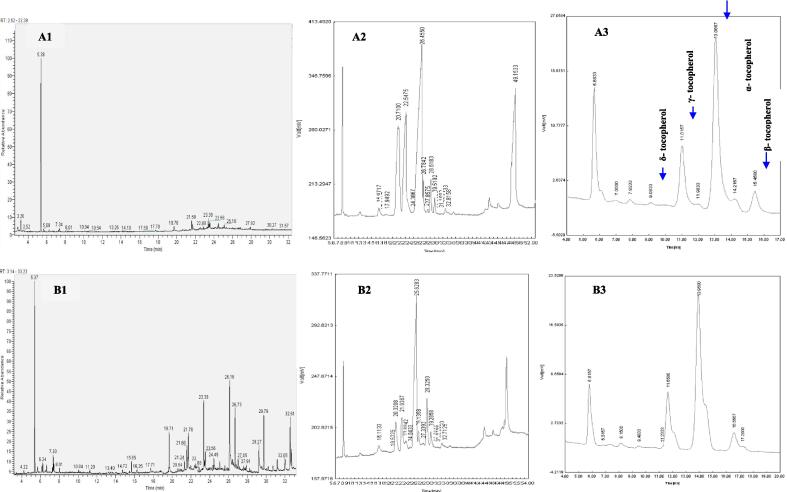
Chromatogram of volatile oxidative (A1 and B1), sterols (A2 and B2) and tocopherols (A3 and B3) compounds of neutralized and industrial bleached sunflower oil, respectively.

The amounts of tocopherols and sterols were determined at raw sunflower oil, industrial condition, HU200SU, HU400, BU400 and BU800 (Table 1 and Fig. 2). The results showed that both of the industrial and ultrasonic bleaching techniques decreased the tocopherol contents of sunflower oil. Industrial, ultrasonic horn (200 W), ultrasonic bath (400 W), ultrasonic horn (400 W), and ultrasonic bath (800 W) bleaching techniques decreased the total tocopherol by 16.80 %, 23 %, 21.3 %, 37.8 %, and 36.6 %, respectively. Accordingly, ultrasonic bath and ultrasonic horn bleaching techniques reduced the tocopherol content of sunflower oil to a greater extent than conventional bleaching techniques. This reduction in tocopherol contents by ultrasonic bleaching technique is related to oxidation reactions and the production of free radicals by ultrasonic waves [11]. The produced free radicals can decompose tocopherols and decline their antioxidant activity in sunflower oil [29]. The level of hydroxylation influences antioxidant activity in food and biological systems. While free radicals can degrade antioxidants, such as phenols, diminishing the bioactivity of food components [29], increased hydroxylation can enhance the antioxidant capacity of certain compounds, including flavonoids [29]. Owing to the decomposition of bioactive compounds after severe radical formation (especially under horn ultrasound) can reduce antioxidant activity.

**Table 1:** Tocopherol and sterol compounds in neutralized, industrialized, and ultrasound-assisted bleaching of sunflower oil.

**Compounds**	**RSU**	**HU_200_SU**	**HU_400_SU**	**BU_400_SU**	**BU_800_SU**	**IBSU**
**α-Tocopherol**	453.82 ± 1.45^a^	353.62 ± 1.75^d^	273.94 ± 1.57^e^	362.20 ± 2.42^c^	278.61 ± 1.87^f^	369.13 ± 2.43^b^
**β-Tocopherol**	32.31 ± 0.34^a^	28.13 ± 0.11^e^	27.97 ± 0.13^f^	28.44 ± 0.18^c^	28.19 ± 0.24^de^	30.17 ± 0.31^b^
**γ-Tocopherol**	93.03 ± 0.74^a^	65.67 ± 0.17^d^	60.92 ± 0.19^e^	66.69 ± 0.13^c^	62.47 ± 0.10^d^	82.51 ± 0.13^b^
**δ-Tocopherol**	9.34 ± 0.06^a^	5.18 ± 0.02^d^	3.31 ± 0.12^f^	5.31 ± 0.05^c^	3.59 ± 0.09^e^	7.80 ± 0.08^b^
**Campesterol**	9.51 ± 0.05^a^	6.00 ± 0.16^d^	5.11 ± 0.08^f^	6.36 ± 0.11^c^	5.45 ± 0.10^e^	8.43 ± 0.07^b^
**Stigmasterol**	144.19 ± 0.23^a^	101.24 ± 1.70^d^	95.67 ± 0.23^f^	104.76 ± 1.65^c^	98.55 ± 0.56^e^	123.90 ± 0.31^b^
**β-Sitosterol**	687.32 ± 0.37^a^	588.34 ± 1.75^d^	567.81 ± 1.62^f^	591.88 ± 2.63^c^	572.24 ± 1.35^e^	620.73 ± 2.76^b^
**Sitostanol**	1.00 ± 0.02^a^	0.85 ± 0.03^c^	0.78 ± 0.01^d^	0.86 ± 0.02^c^	0.68 ± 0.01^e^	0.91 ± 0.04^b^
**Δ7-avenasterol**	1.00 ± 0.16^a^	0.72 ± 0.19^b^	0.64 ± 0.10^c^	0.77 ± 0.15^b^	0.65 ± 0.16^c^	0.94 ± 0.06^a^
**Δ7-stigmastenol**	48.91 ± 0.56^a^	38.31 ± 0.15^d^	36.15 ± 0.51^f^	39.33 ± 0.22^c^	37.60 ± 0.21^e^	43.92 ± 0.15^b^
**Δ5-avenasterol**	253.84 ± 1.76^a^	220.19 ± 0.95^c^	209.87 ± 1.76^e^	221.74 ± 1.07^c^	213.19 ± 1.30^d^	232.63 ± 1.55^b^

** Each data represents means of triplicates ± SD in same raw.

***ND means not detected.

**Fig. 3:**
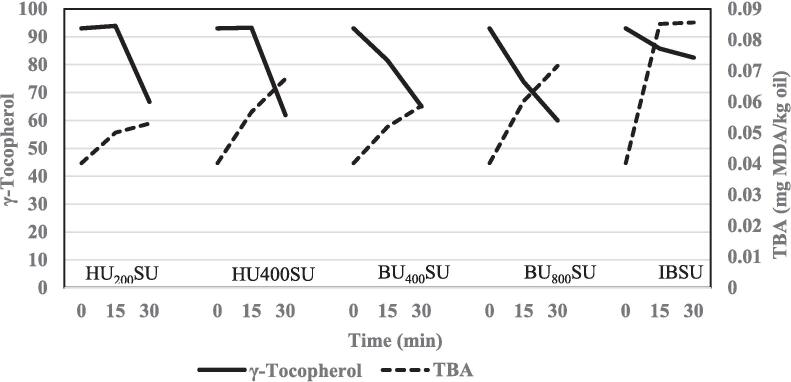
Relationship between γ-tocopherol degradation and TBA value of sunflower oil samples during bleaching. HU_200_SU: ultrasonic horn (200 W), HU_400_SU: ultrasonic horn (400 W), BU_400_SU: ultrasonic bath (400 W), BU_800_SU: ultrasonic bath (800 W), IBSU: industrial bleaching, TBA: thiobarbituric acid, and MDA: malondialdehyde.

The ultrasonic bath bleaching technique (400 W) showed a lower reduction in total tocopherol contents than that of the ultrasonic horn bleaching technique (400 W). This may be due to the production of lower amounts of free radicals in the ultrasonic bath bleaching technique than in the ultrasonic horn bleaching technique, which has the same power. In the industrial bleaching process, the highest and lowest reduction of tocopherol belonged to α-tocopherol and β-tocopherol, respectively. In the ultrasonic bath and ultrasonic horn bleaching techniques, γ-tocopherol showed the highest reduction, while β-tocopherol showed the lowest reduction. The relationship between γ-tocopherol degradation and TBA value during sunflower oil bleaching by industrial and ultrasonic techniques is presented in Fig. 3. The reduction in γ-tocopherol content was in line with increasing TBA value (Fig. 3). Sterol, as well as tocopherol, was sensitive to both the industrial and ultrasonic bleaching techniques (Table 1 and Fig. 2). This reduction in sterols during bleaching can be the result of isomerization, adsorption, hydrolysis, dehydration, and esterification which reflects an enhancement in non-polar components and terpenes [30].

Industrial, ultrasonic horn (200 W), ultrasonic bath (400 W), ultrasonic horn (400 W), and ultrasonic bath (800 W) bleaching techniques decreased the total sterol contents by 9.98 %, 16.60 %, 15.73 %, 20.04 %, and 18.96 %, respectively. Similar to tocopherol results, ultrasonic bath and ultrasonic horn bleaching techniques decreased the total sterol content of sunflower oil to a greater extent than industrial bleaching techniques. Among the ultrasonic techniques, sterols showed the highest stability during bleaching by ultrasonic bath technique (400 W). The reduction percentage of sterols during bleaching by industrial and ultrasonic techniques was lower than those of tocopherols. Accordingly, tocopherols were more sensitive to degradation than sterols during bleaching by industrial and ultrasonic techniques.

In the industrial bleaching technique, Δ7-Avenasterol and campesterol showed the highest and lowest stability, respectively. In the ultrasonic techniques, sitostanol showed the highest stability in the ultrasonic bath technique, while in the ultrasonic horn technique, Δ5-avenasterol showed the highest stability. Also, campesterol showed the lowest stability during bleaching by both the ultrasonic bath and horn techniques.

### 3.3 Effect of ultrasonic bath and horn techniques on the fatty acid composition

The fatty acid composition of sunflower oil samples bleached by industrial and ultrasonic techniques was similar to that of raw sunflower oil samples (Table 2). Accordingly, bleaching by industrial, ultrasonic bath, and ultrasonic techniques did not significantly affect the fatty acid composition of sunflower oil. Similar results were observed for olive oil [14], [27], soybean oil [11], and rice bran oil [31].

**Table 2:** Fatty acid profile in neutralized, industrialized and ultrasound-assisted bleaching of sunflower oil.

**Fatty acid (%)**	**RSU**	**HU200SU**	**HU400SU**	**BU400SU**	**BU800SU**	**IBSU**
14:0	0.11 ± 0.04	0.10 ± 0.03	0.12 ± 0.06	0.10 ± 0.08	0.11 ± 0.03	0.11 ± 0.04
16:0	7.30 ± 1.80	7.27 ± 1.24	7.29 ± 0.97	7.25 ± 1.03	7.26 ± 1.03	7.30 ± 1.38
16:1ω_7_	0.08 ± 0.00	0.06 ± 0.01	0.08 ± 0.02	0.07 ± 0.01	0.09 ± 0.04	0.08 ± 0.02
18:0	4.58 ± 0.84	4.50 ± 1.43	4.54 ± 0.87	4.52 ± 1.0	4.59 ± 1.23	4.55 ± 1.21
18:1 ω_9_	27.29 ± 2.67	27.26 ± 2.59	27.24 ± 1.00	27.27 ± 1.95	27.31 ± 1.13	27.32 ± 1.95
18:2 ω_6_	50.52 ± 2.59	51.61 ± 1.93	50.53 ± 1.68	50.57 ± 2.81	50.60 ± 1.33	50.54 ± 2.81
18:3 ω_3_	0.25 ± 0.02	0.30 ± 0.04	0.26 ± 0.07	0.28 ± 0.04	0.25 ± 0.08	0.23 ± 0.04
20:0	0.16 ± 0.01	0.13 ± 0.02	0.14 ± 0.04	0.13 ± 0.01	0.18 ± 0.04	0.15 ± 0.01

** Each data represents means of triplicates ± SD.

***ND means not detected.

### 3.4 Effect of ultrasonic bath and horn techniques on the volatile compounds

Some of the initial volatile compounds generated mainly through biochemical pathways are responsible for the palatable sensory characteristics of the oil. Still, off-flavours are produced through autoxidation and oxidation of unsaturated lipids [32]. Oxidative deterioration of sunflower oil during bleaching can be determined by measuring the number and concentration of the produced volatile compounds. The total amounts of volatile compounds were increased after bleaching sunflower oil using industrial and ultrasonic techniques (Table 3). The activated bleaching clay can decompose lipid hydroperoxides and form volatile compounds [20]. Some volatile compounds, such as camphene, which was absent in raw sunflower oil, were detected in the sunflower oil after bleaching by industrial and ultrasonic techniques. Also, sabinene and 1, 8- cineol were detected in sunflower oil bleached by ultrasonic bath and horn techniques, while they were absent in the raw and bleached sunflower oil by industrial technique.

**Table 3:** Volatile oxidative compounds in different power of horn and bath ultrasonic.

**Compounds**	**Odor detected**	**RSU**	**HU_200_SU**	**HU_400_SU**	**BU_400_SU**	**BU_800_SU**	**IBSU**
**Xylene**	Benzene	0.50 ± 0.03	0.71 ± 0.08	0.78 ± 0.02	0.64 ± 0.06	0.76 ± 0.02	0.62 ± 0.04
**α-Pinene**	Minty and pine scent	25.07 ± 0.04	56.34 ± 0.09	81.44 ± 0.06	51.91 ± 0.09	76.33 ± 0.05	42.14 ± 0.08
**Camphene**	Woody	−	0.79 ± 0.12	1.15 ± 0.09	0.56 ± 0.08	0.89 ± 0.04	0.26 ± 0.00
**Sabinene**	Warm, oily-peppery, woody-herbaceous and spicy odor of moderate to poor tenacity	−	0.26 ± 0.08	0.76 ± 0.12	0.12 ± 0.04	0.69 ± 0.01	−
**β-Myrcene**	Spicy, earthy and musky	0.58 ± 0.01	0.71 ± 0.09	0.98 ± 0.02	0.69 ± 0.05	0.78 ± 0.04	0.66 ± 0.02
**n-Decane**	Gasoline	0.45 ± 0.02	0.55 ± 0.07	0.78 ± 0.05	0.47 ± 0.04	0.71 ± 0.04	0.41 ± 0.01
**p-cymene**	Harsh chemical, and woody like	0.52 ± 0.01	0.70 ± 0.01	0.101 ± 0.06	0.65 ± 0.04	0.89 ± 0.06	0.61 ± 0.02
**Limonene**	Fresh, sweet	1.44 ± 0.01	1.69 ± 0.07	2.67 ± 0.08	1.34 ± 0.08	2.42 ± 0.05	1.71 ± 0.02
**1,8-Cineol**	Eucalyptus-like odor	−	0.38 ± 0.08	1.07 ± 0.05	0.16 ± 0.04	0.99 ± 0.02	−
**2(E)-Octenal**	Fatty-nutty	2.02 ± 0.02	2.96 ± 0.08	4.23 ± 0.09	2.55 ± 0.08	3.71 ± 0.16	2.47 ± 0.03
**n-Tetradecane**	Gasoline-like to odorless	0.78 ± 0.04	0.89 ± 0.11	1.71 ± 0.08	0.73 ± 0.06	1.31 ± 0.09	0.69 ± 0.04
**Pentanal**	Fermented, bready, fruity, nutty, berry	1.73 ± 0.02	2.43 ± 0.09	7.02 ± 0.11	2.67 ± 0.09	5.33 ± 0.13	2.47 ± 0.02
**Heptadecane**	Fuel-like	0.61 ± 0.06	5.12 ± 0.17	8.73 ± 0.12	5.68 ± 0.13	8.21 ± 0.14	0.89 ± 0.02
**Pentadecanol**	A faint odor of alcohol	0.09 ± 0.00	0.78 ± 0.09	1.60 ± 0.08	0.83 ± 0.09	1.49 ± 0.04	0.23 ± 0.01
**2,3-Octanedione**	Dill-type odor	2.28 ± 0.03	0.93 ± 0.08	1.97 ± 0.07	0.85 ± 0.09	1.24 ± 0.03	2.21 ± 0.002
**Nonanal**	Fatty, waxy, citrus	4.52 ± 0.08	7.71 ± 0.19	6.93 ± 0.04	7.31 ± 0.19	6.81 ± 0.05	8.23 ± 0.05
**n-Hexadecane**	Gasoline	3.12 ± 0.03	4.18 ± 0.13	8.25 ± 0.08	3.94 ± 0.13	7.87 ± 0.09	3.61 ± 0.02
**Hexanal**	Fatty, pungent, grassy	9.52 ± 0.08	10.17 ± 0.19	15.93 ± 0.16	11.64 ± 0.09	13.03 ± 0.07	9.93 ± 0.08
**Cyclooctene**	Camphoraceous	1.00 ± 0.01	1.23 ± 0.09	3.49 ± 0.04	1.78 ± 0.11	3.98 ± 0.03	1.17 ± 0.01
**(2E,4E)-deca-2,4-dienal**	Deep fried,	1.37 ± 0.05	4.93 ± 0.09	9.27 ± 0.06	3.98 ± 0.06	8.01 ± 0.03	3.15 ± 0.08
**2(Z)-Heptenal**	Fishy, sweet	0.98 ± 0.02	1.65 ± 0.06	5.63 ± 0.06	2.44 ± 0.06	4.88 ± 0.04	1.90 ± 0.08
**β-Pinene**	Woody-green, pine-like	ND	ND	ND	ND	ND	ND

** Each data represents means of triplicates ± SD.

***ND means not detected.

The amount of total volatile compounds was as follow: ultrasonic horn (400 W)> ultrasonic bath (800 W)> ultrasonic horn (200 W) > ultrasonic horn (400W). Accordingly, the ultrasonic bath technique (400 W) showed fewer total volatile compounds than the ultrasonic horn technique (400 W). This can be due to the lower oxidation rate of sunflower oil during bleaching by the ultrasonic bath technique than that of the ultrasonic horn technique at the same power. The total volatile compounds increased by increasing power in both the ultrasonic bath and ultrasonic horn techniques. Thus, higher ultrasound powers make the sunflower oil more susceptible to oxidative degradation. The highest increase in the industrial bleaching technique was observed for pentadecanol, followed by (2E,4E)-deca-2,4-dienal, and 2(Z)-heptenal. In the ultrasonic bath and horn technique, the highest increase was observed for pentadecanol, followed by heptadecane and (2E,4E)-deca-2,4-denial.

## 4 Conclusion

This study aimed to compare the effect of bath and horn ultrasound-assisted bleaching of sunflower oil at different powers on the degradation of tocopherols and sterols, production of volatile substances, and oxidation indices with that of the industrial bleaching technique. In both the ultrasonic bath and ultrasonic horn techniques, sunflower oil samples bleached at higher power showed higher TBA values than those bleached at lower power. Besides, the TBA values of sunflower oil samples bleached by the ultrasonic bath technique (400 W) were lower than those bleached by the ultrasonic horn technique at the same power. Ultrasonic horn and ultrasonic bath bleaching techniques reduced the total tocopherol and sterol contents of sunflower oil to a greater extent than that of the industrial bleaching techniques. In addition, the tocopherol and sterol compounds were more sensitive to degradation during bleaching sunflower oil by the ultrasonic horn technique than the ultrasonic bath technique. Bleaching sunflower oil by industrial and ultrasonic techniques did not significantly affect the fatty acid composition of sunflower oil. Sunflower oil samples bleached by ultrasonic bath showed lower amounts of total volatile compounds than those bleached by ultrasonic horn technique at 200 and 400 W and ultrasonic bath technique at 800 W. In general, the bioactive compounds of sunflower oil were less sensitive to degradation during bleaching by the ultrasonic bath technique than by the ultrasonic horn technique. Also, the rate of sunflower oil oxidation during bleaching by the ultrasonic bath technique was lower than that of the ultrasonic horn technique. The results of this study can help oil industry manufacturers choose appropriate novel techniques for oil bleaching.

## CRediT authorship contribution statement

**Mehran Sayadi:** Methodology, Funding acquisition. **Elahe Abedi:** Writing – review & editing, Writing – original draft, Resources, Project administration, Methodology, Investigation. **Malihe Keramat:** Writing – review & editing, Writing – original draft, Investigation.

## Declaration of competing interest

The authors declare that they have no known competing financial interests or personal relationships that could have appeared to influence the work reported in this paper.
